# Exploration of interoceptive capabilities in avoidant/restrictive food intake disorder and anorexia nervosa

**DOI:** 10.1186/s40337-023-00914-9

**Published:** 2023-10-23

**Authors:** Nandini Datta, James D. Lock

**Affiliations:** Department of Psychiatry and Behavioral Sciences, Stanford University School of Medicine, 401 Quarry Road, Stanford, CA 94305 USA

**Keywords:** Interoception, Adolescent, ARFID, Anorexia nervosa

## Abstract

**Objective:**

This proof-of-concept study explores the role of aberrant interoception as a possible mechanism underlying restrictive eating symptoms in avoidant/restrictive food intake disorder (ARFID) compared to anorexia nervosa (AN) and healthy comparisons (HC).

**Method:**

We report preliminary normative adolescent interoceptive data in HCs (n = 100) compared to adolescents with ARFID (n = 30) and AN (N = 23). Adolescents (12–18) participated in a one-time virtual visit to assess heartrate guessing accuracy (interoceptive accuracy), correlation between confidence in heartrate guess and accuracy (interoceptive awareness), and self-reported interoception (interoceptive sensibility).

**Results:**

HC adolescents had comparable interoceptive outcomes relative to published adult norms, consistent with existing literature. Data suggest that adolescents with ARFID have poor heartbeat guessing accuracy and experience challenges deciphering interoceptive signals, possibly contributing to symptoms. While adolescents with AN have *greater* heartbeat guessing accuracy, they cite difficulty trusting body cues, perhaps contributing to their lack of confidence in interoceptive cue detection.

**Conclusions:**

Preliminary results reflect differences in interoception between the three groups.

## Abstract (plain language summary)

In this study, we wanted to understand how individuals with restrictive eating disorders, like avoidant/restrictive food intake disorder (ARFID) and anorexia nervosa (AN), sense and respond to different signals in their body, such as heartbeat. This process is called interoception. Difficulty sensing body signals can lead to various mental health disorders, such as eating disorders. We asked adolescent participants without an eating disorder and adolescents with ARFID and AN to guess their heartbeat while wearing a finger pulse oximeter, to track interoceptive accuracy (or how accurate their heartbeat guesses were). We also measured their self-reported sense of interoception and overall metacognitive awareness of their ability to accurately interpret body signals. Our data replicate findings in existing healthy teenagers. For teens with eating disorders, those with ARFID had difficulty with interoceptive accuracy and listening to their bodies, but they had good awareness overall of their accuracy. Teens with AN had better accuracy but were more unsure about trusting their body and body signaling. Overall, our initial findings suggest that how individuals with eating disorders sense, interpret, and respond to body signals might help explain their eating habits. More research is needed in this area.

## Background

Avoidant/restrictive food intake disorder (ARFID) is an eating disorder (ED) characterized by restrictive eating habits resulting in nutritional deficiencies and/or psychosocial impairment [1, 2]. Unlike anorexia nervosa (AN), there are no accompanying concerns about body shape or weight; rather, restrictive eating is driven by low appetite, severe “picky” eating due to sensory sensitivity, or fear of aversive consequences [3]. ARFID has prevalence rates from a specialized eating disorder service at 5–22%, and from non-clinical samples at 0.3–15.5% [4, 5], making it a serious mental health concern. Limited published studies have investigated the underlying pathophysiology of ARFID, which is necessary to better understand this debilitating disorder and inform treatments. Both ARFID and AN share sensory disturbances in gastric signaling, such as post-prandial discomfort and early satiety [6]. To that end, one mechanism implicated in AN that may impact symptom development in ARFID is aberrant interoception, or the perception and response to changes in bodily signals (such as heartbeat detection or satiety cues) [7].

Interoceptive experiences motivate behaviors that promote a return to homeostasis and are strongly linked to emotional and cognitive processing [8, 9]. Interoceptive signaling is shaped by neural predictions rooted in prior experiences and beliefs about bodily states. When these predictions are mismatched with the interoceptive signal being experienced, the resulting ‘prediction error’ putatively underlies various psychopathology, including eating disorders [6, 10]. Current cardiac interoceptive accuracy norms in healthy adolescents are comparable with those published in adults [11], while self-reported interoception continues to develop throughout adolescence [12, 13]. Domains such as trusting one's body appear to reduce as an individual moves through adolescence to adulthood [13]. Multidimensional batteries of interoception in a large sample of adolescents are lacking in the literature.

In AN, some studies have found that aberrant interoception emerges as a consequence of prolonged malnutrition, often lasting after weight restoration [14]. However, inconsistent reports of interoceptive functioning in AN across development require additional investigation [15–18]. The majority of these studies have been done in adults, with limited evidence of interoception in adolescents with AN. Studies of interoception in adolescent eating disorders transdiagnostically have found self-report differences in domains of interoception, where adolescents with EDs report greater body trust than adults with EDs [19]. Further, a network analysis demonstrated similar interoceptive capabilities between adults and adolescents with EDs, with lack of body trust being the most associated with eating disorder symptoms [20]. In a sample focused on children and adolescents, one study found no differences in self-reported interoception between AN and ARFID [21]. Additional research on interoception in adolescents with AN is needed to help shed light on these processes during a crucial period of development.

There are no systematic, multi-method data exploring interoceptive capabilities in adolescents with ARFID [7, 22, 23]. Clinically, individuals with ARFID report uncomfortable somatic experiences after eating, such as gastroesophageal reflux or gastric discomfort with satiety [24]. Thus, it is possible that disruptions in interoception may be a transdiagnostic feature of restrictive eating [21, 25]. In a review of interoceptive processes in eating disorders [6], Khalsa and colleagues report that while youth with ARFID may have elevated sensitivity in response to gastric signaling, whether this is driven by heightened sensitivity from external cues (e.g., smells and sounds of other eating) versus sensitivity to internal signaling (e.g., hunger cues) is unknown [26].

This proof-of-concept investigation sought to explore interoceptive differences between a healthy adolescent sample and two restrictive eating disorder samples: ARFID and AN. We also conducted a preliminary descriptive investigation on interoceptive differences between ARFID subgroups (sensory sensitivity, low appetite, fear of aversive consequences). Based on prior literature, we anticipated that adolescent norms would replicate existing adolescent data [27] and that youth with ARFID would demonstrate poorer interoceptive capabilities compared to HCs. Based on inconsistent literature on AN interoception, we anticipated that individuals with AN would have greater interoceptive accuracy than HC and ARFID but report less confidence and lower self-reported interoceptive capabilities. We had no a priori hypotheses about ARFID subgroup differences. We assessed interoception using a virtual heartbeat detection task (to probe for *interoceptive accuracy*, or how accurately one detects internal sensations such as heartbeat), confidence in heartbeat guesses (confidence scores correlated with accuracy scores provide a metric for *interoceptive awareness* or metacognitive awareness of interoceptive capabilities), and a self-report questionnaire on interoceptive abilities (*interoceptive sensibility*, or one’s subjective judgment of their own perception of internal body signaling).

## Methods

### Participants

This exploratory study was conducted virtually during the COVID-19 pandemic and approved by Stanford University’s Institutional Review Board. Participants who were 18 years of age provided consent; participants under 18 years of age provided assent, and their parents provided consent. Clinical participants were actively recruited from clinics, hospitals, schools, and through online advertisements. HC participants were recruited through online advertisements and schools. All participants were between the ages of 12–18. Clinical participants needed to have a diagnosis of ARFID or AN. Healthy comparison participants needed to be free of any psychiatric diagnoses. All participants completed a preliminary screen to determine eligibility. A larger sample of healthy control adolescents was collected (n = 100) compared to the clinical samples (n = 20–30) to replicate existing adolescent norms. Descriptive and clinical data for each group is provided in Table 1.

**Table 1:** Demographics and clinical features

	ARFID	AN	HC
Age M (SD)^†^	14.3 (1.9)	15.2 (1.9)	15.1 (1.8)
Sex (% female)	56.7%	95.2%	65.7%
Sex (% male)	43.3%	4.8%	34.3%
*Gender*			
% girls	46.7%	90.5%	56.6%
% boys	50%	4.7%	42.4
Prefer not to say	3.3%	4.8%	1%
Other	0%	0%	2%
Non-binary	3.3%	0%	0%
White	66.7%	57.1%	45.5%
Asian	3.3%	19%	22.2%
Black	–	–	4%
NHPI^††^	–	–	1%
Multi-racial	16.7%	19%	15.2%
Hispanic	16.7%	9.5%	12.1%
*Comorbidity*			
Major depressive disoder	23%	23%	–
Generalized anxiety disorder	20%	14%	–
Anxiety disorder NOS	3.33%	4.7%	–
Autism spectrum disorder	3.33%	–	–
Attention-deficit/hyperactivity disorder	20%	–	–
Psychotropic medication (% taking)	56.7%	23.8%	–
%EBW*	93.4 (14.2)	85.3 (15.3)	107.6 (17)
EDE-global	0.6 (0.9)	2.8 (1.6)	0.8 (1.0)
NIAS** sensory sensitivity profile	11.6 (3.5)	6.5 (4.1)	3.2 (3)
NIAS low appetite profile	8.9 (4.1)	4.7 (4.3)	2.7 (2.5)
NIAS fear profile	4.7 (4.0)	4 (3.8)	1.1 (1.9)

^†^M, Mean; SD, Standard deviation; ^††^NHPI, Native Hawaiian and Pacific Islander; *EBW, Expected Body Weight; ** NIAS, Nine Item Avoidant/Restrictive Food Intake Disorder (ARFID) Screener—of note, clinical cut-offs for the ARFID screener subscales are as follows: (Sensory Sensitivity Profile ≥ 10 & EDE-Q < 2.3; Low Appetite Profile ≥ 9 & EDE-Q < 2.3; and Fear Profile ≥ 10 & EDE-Q < 2.3) [31]

### Study procedures

All study visits were done virtually since this study was completed during the COVID-19 pandemic. Before their visit, all participants were mailed a standard finger pulse oximeter to use during the heartbeat task (described below). A one-time 45-min Zoom appointment was scheduled to complete questionnaires, a diagnostic interview, and behavioral measures of interoception conducted by the first author.

### Measures

We measured interoception via three domains, listed below, that are consistent with prior descriptions of interoception in the current literature:

*Interoceptive accuracy* [7, 27]: The ability to accurately detect internal body signals such as heart rate or hunger/fullness cues.

*Interoceptive awareness* [27]: The association between one’s confidence in or perception of their interoceptive accuracy and their actual interoceptive accuracy. In other words, a metacognitive awareness of interoceptive accuracy.

*Interoceptive sensibility* [7]: One’s own perception (or self-report) of how tuned in they are to interoceptive signaling.

Cardiac interoception, as probed by heartbeat tasks, has been found to correlate with other measures of interoception interrogating other organ systems, such as sensitivity to gastrointestinal cues [7, 27], relevant for eating disorders. The tasks used to probe each domain are outlined below:

*Demographics*: Participants provided basic demographic information, including age, pubertal status, sex/gender, height, weight, ethnicity, comorbidities, and current medications. Percent of expected body weight (%EBW) was calculated using CDC median BMI for each participant’s age and sex [28].

*Eating disorder assessment-5* [29]. The EDA-5 is a semi-structured interview assessing the presence of a feeding/ eating disorder according to current DSM-5 criteria. This measure was used to confirm ED diagnosis for participants in the present study. A PhD-level clinician trained in the EDA-5 administered this assessment.

*Nine-item ARFID screen* [30]: The NIAS is a nine-item self-report questionnaire assessing eating habits in ARFID clinical presentations (fear, sensory sensitivity, and/or low appetite). Higher scores reflect greater clinical severity.

ARFID subgroups were determined by score cut-offs on the NIAS, established in the literature [31]. Specifically, to meet criteria for the sensory sensitivity subgroup, scores needed to be ≥ 10 (sensitivity = 0.97, specificity = 0.63); to meet criteria for the low appetite subgroup, scores needed to be ≥ 9 (sensitivity = 0.86, specificity = 0.70); and to meet criteria for the fear of aversive consequences subgroup, scores needed to be ≥ 10 (sensitivity = 0.68, specificity = 0.89). The subgroups are defined as follows:

*Sensory sensitivity*: sensitivity to sensory characteristics of food, such as texture, temperature, or taste.

*Low appetite*: having a pervasive lack of interest in eating or foods.

*Fear of aversive consequences*: worry about an aversive consequence of eating, such as getting ill, choking, or vomiting.

*Eating disorder examination-questionnaire* [32]: The EDE-Q is a 32-item self-report questionnaire assessing eating disorder symptomatology over the past 28 days. Greater scores reflect greater severity of eating psychopathology driven by shape and weight concerns.

*Heartbeat Tracking Task (Interoceptive Accuracy)* [27, 33–35]. Participants silently counted their heartbeat (without manually checking) over six trials using randomly presented time windows of 25, 30, 35, 40, 45, and 50 s. Accuracy scores were calculated using: 1 − (|nbeats_real_ − nbeats_reported_|)/((nbeats_real_ + nbeats_reported_)/2), averaged over six trials, producing one accuracy score per participant. This score varies between 0 and 1, where scores of 1 indicate 100% accuracy in detecting one’s heartbeat.

*Confidence rating (interoceptive awareness)* [27]*.* After each heartbeat trial, participants rated their confidence in the perceived accuracy of their response, using a continuous visual analog scale from 0 (total guess/no heartbeat awareness) to 10 (complete confidence/fully aware of heartbeat). An index of interoceptive awareness was computed using correlations between confidence ratings and accuracy scores, described above. Greater correlations reflect better interoceptive awareness.

*The multidimensional assessment of interoceptive awareness-2 (MAIA-Y) (interoceptive sensibility)* [13, 36]. The MAIA-Y is a 37-item questionnaire measuring domains of interoceptive awareness, where higher scores indicate greater attention to body signals. There are eight subscales: “*noticing*” (explores awareness of body sensations, i.e., “I can tell when I am uncomfortable in my body”); “*not-distracting*” (how tuned in on is to body sensations of discomfort, without using distraction); “*not-worrying*” (index of participants’ ability to not become emotionally reactive to negative physical sensations, i.e., “When I feel pain in my body I become upset”); “*attention-regulation*” (explores the capacity to regulate attention with multiple competing sensory stimuli, i.e., “I can focus on my entire body when I try"); “*emotional-awareness*” (explores the ability to be aware of the relationship between body-states and emotions, i.e., “I can feel how my body changes when I am angry”); “*self-regulation*” (explores the use of attention to body-states to regulate psychological distress, i.e., “I can use my breath to help me calm down and relax”); “*body-listening*” (index of attending to body sensations for insight, i.e., “I listen for clues from my body about my emotions”); and “*trusting*” (how much body cues are experienced as trustworthy, i.e., “I trust the way my body feels” or “I feel my body is a safe place”). Each subscale has a score from 0 to 5, where greater scores reflect more regulated and adaptive attention to body signaling or interoceptive cues.

### Statistical approach

Data met assumptions of normality, and no outliers were identified in the dataset using visual examination of skewness and kurtosis and assessment of scatterplots to confirm the use of a GLM model. The pattern of missing data were assessed prior to analyses and identified to be missing completely at random (MCAR), by Little’s MCAR test.

We conducted exploratory group comparisons to examine between-group effects. Considering the pilot nature of this study and small sample sizes of the clinical groups, we report partial eta square for the overall model, Hedge’ *g* effect size estimates (ES) for pairwise comparisons, and Confidence Intervals [CI] for correlations [37–39]. For interpretation: η^2^ of 0.01 = small effect, 0.06 = medium effect and 0.14 = large effect. For Hedge’s *g*, 0.2 = small effect, 0.5 = medium effect, and 0.8 = large effect [40]. Analyses were completed using SPSS Version 27. A one-way between-subjects ANCOVA was conducted to compare interoceptive accuracy. A one-way ANCOVA was conducted to determine a statistically significant difference between groups (AN, HC, ARFID) on interoceptive sensibility (self-report subscales on the MAIA-Y) controlling for sex and %EBW. %EBW was added as a covariate in analyses since it varied between groups and can be implicated in heartbeat detection [41, 42]. Sex assigned at birth was also entered as a covariate in analyses as there have been reported differences in heartrate counting tasks between males and females across development [43]. Partial Pearson’s correlations controlling for sex and %EBW were conducted to assess the degree of correlation between a participant’s accuracy score and their level of confidence in their guess. Higher correlations reflect a greater awareness of internal body sensations, such as heartbeat. These data are not publicly available due to privacy and ethical restrictions.

**Fig. 1:**
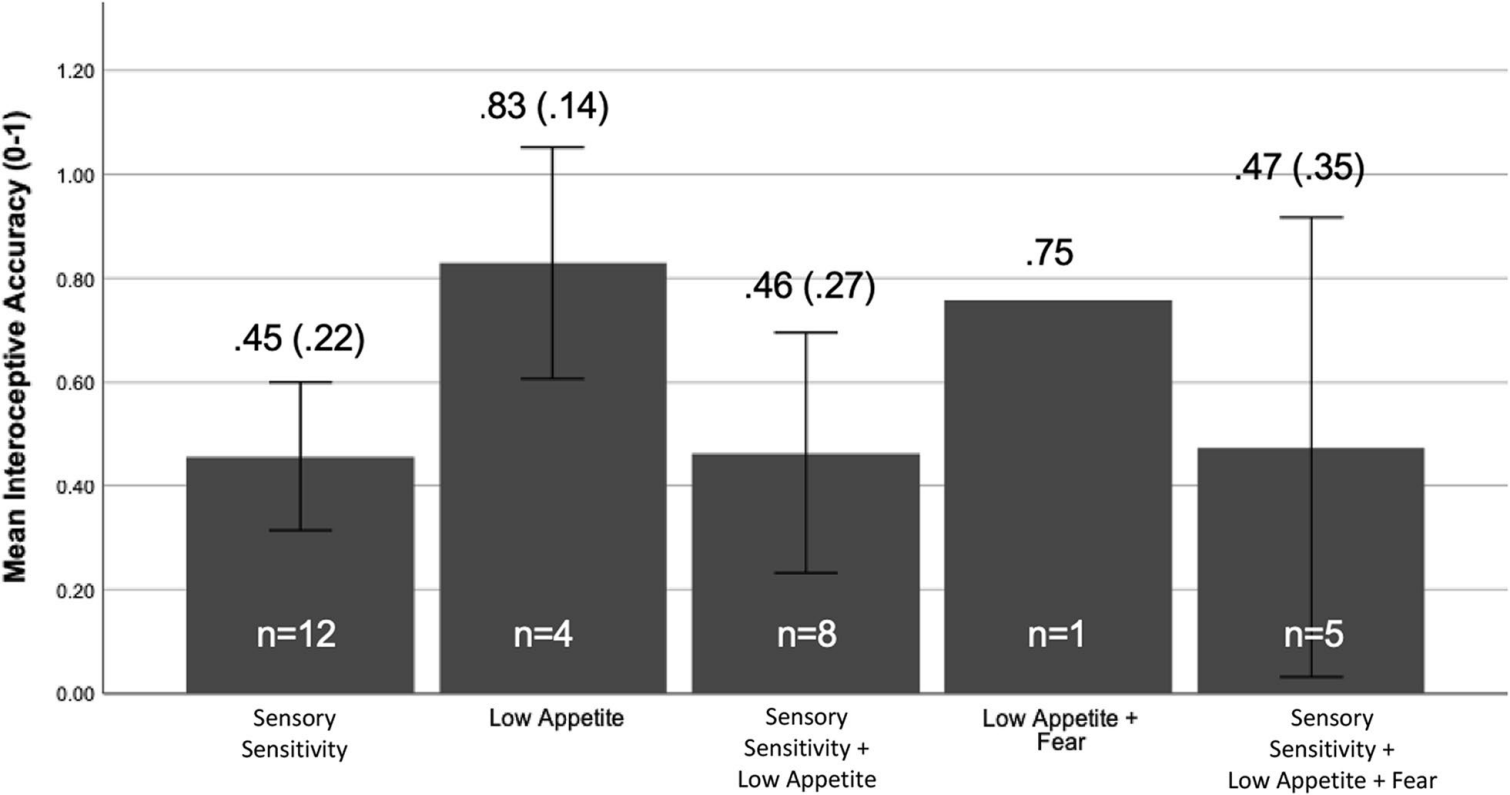
Interoceptive accuracy by ARFID subgroup

**Fig. 2:**
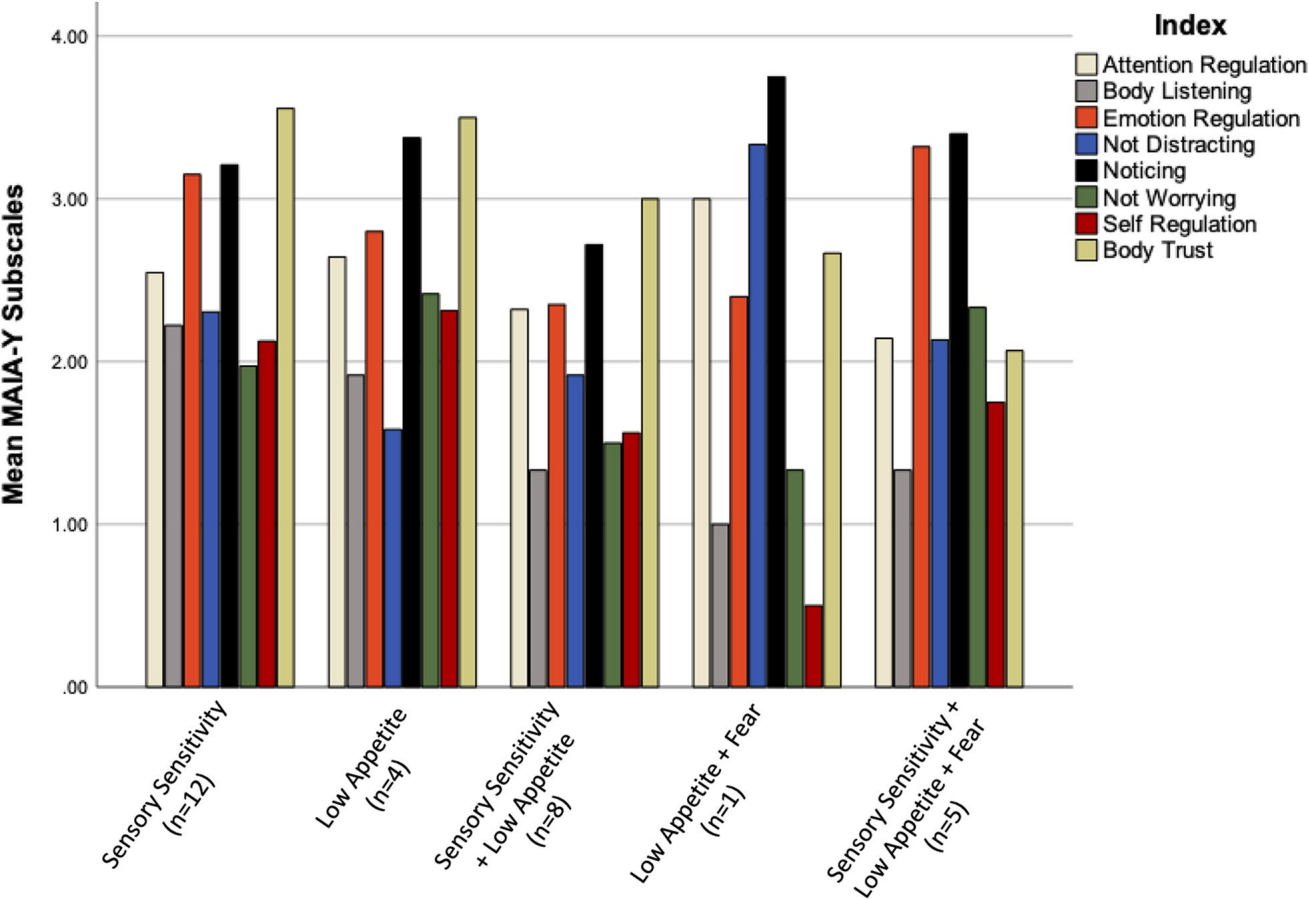
Interoceptive sensibility by ARFID subgroup

We also descriptively explored interoceptive differences between ARFID subgroups. These differences are depicted via bar graphs (Figs. 1 and 2) and a table (Table 3).

## Results

### Interoceptive accuracy

A one-way ANCOVA was conducted to determine a statistically significant difference between groups (AN, HC, ARFID) on interoceptive accuracy (heartbeat detection) controlling for sex and %EBW. We found a significant effect of group on interoceptive accuracy F(2, 148) = 4.71, η^2^ = 0.061 (medium effect size). For pairwise comparisons, individuals with ARFID had lower interoceptive accuracy than HC participants (M = 0.66, SD = 0.28], CI [0.02–0.29], Hedge’s *g* = 0.58, but not AN participants.

Interoceptive accuracy (Table 2) was greater for individuals with AN (M = 0.69, SD = 0.11) relative to those with ARFID (M = 0.50, SD = 0.27; CI [−0.37 to −0.001], Hedge’s *g* = 0.86; F(2,149) = 4.71, but not HC participants.

**Table 2:** Main Interoceptive Outcomes

Interoceptive domain M (SD)	ARFID n = 30	AN n = 22	HC n = 100	SS	F	95% CI	Hedge’s *g*
Interoceptive accuracy	0.50 (0.27)	0.69 (0.11)	0.66 (0.28)	0.62	4.71	− 0.37 to 0.001	ARFID < AN = 0.86
						− 0.22 to 0.29	ARFID < HC = 0.47
Interoceptive awareness (Pearson’s *r*[95% CI])	0.40 [0.10 to 0.68]	0.13 [− 0.31 to 0.54]	0.35 [0.16–0.51]	–	–	–	–
MAIA-Y noticing	3.14 (0.96)	2.73 (0.86)	3.36 (0.84)	5.49	3.70	0.07–1.2	AN < HC = 0.74
MAIA-Y not distract	2.10 (1.03)	1.87 (0.93)	2.54 (0.96)	7.97	4.24	0.03–1.30	AN < HC = 0.80
MAIA-Y not worry	1.94 (0.84)	2.19 (0.83)	2.50 (0.85)	6.59	4.71	0.11–1.00	ARFID < HC = 0.65
MAIA-Y attention regulation	2.40 (1.02)	2.30 (0.81)	2.76 (0.91)	4.38	2.59		All < 0.2
MAIA-Y emotion regulation	291 (1.10)	2.65 (1.03)	2.99 (0.93)	1.60	0.83		All < 0.2
MAIA-Y self-regulation	1.91 (1.02)	1.90 (1.34)	2.56 (0.95)	11.59	5.91	0.12–1.18	ARFID < HC = 0.70
						0.02–1.34	AN < HC = 0.58
MAIA-Y body listening	1.76 (1.20)	1.25 (0.99)	2.17 (1.01)	12.40	5.72	0.24–1.62	AN < HC = 0.92
MAIA-Y body trust	2.99 (1.10)	1.82 (1.38)	3.77 (0.98)	53.64	24.6	− 1.26 to 2.6	AN < HC = 1.84
						− 1.91 to 0.41	AN < ARFID = 0.95
						− 1.35 to 0.23	ARFID < HC = 0.78

Hedge’s g, 0.2 = small effect; 0.5 = medium effect; 0.8 = large effect; Pearson’s *r*, 0.1 = small effect; 0.3 = medium effect; > 0.5 = large effect

### Interoceptive awareness

Interoceptive awareness was greater for HC (*r*(98) = 0.35 (medium effect), CI [0.16–0.52]) and ARFID participants (*r*(28) = 0.40 (medium effect), CI [0.10–0.68]) relative to AN participants (*r*(20) = 0.13 (small effect), CI [−0.31 to 0.54]).

### Interoceptive sensibility

We found a significant effect of group on specific MAIA-Y subscales, detailed below.

*ARFID.* For pairwise comparisons, participants with ARFID reported lower scores on the “Not Worry” subscale, reflecting more difficulty not becoming emotionally reactive to negative physical sensations (F(2, 149) = 4.71) compared to HC (Hedge’s *g* = 0.65, CI [−0.11 to 0.1.00]) but not AN. Participants with ARFID also had lower scores on the “Self-Regulation” and “Body Trust” subscale compared to HC, but not AN, indicating greater difficulty in using attention to body states to self-regulate physiological distress and less trust in body sensations (F(2,149) = 5.91; Hedge’s g = 0.70–0.78, CI [−0.12 to 1.18]).

*AN.* Participants with AN reported lower scores than HC participants but not ARFID on the “Noticing” subscale (Hedge’s *g* = 0.74), the “Not Distract” subscale (Hedge’s *g* = 0.58), and the “Self-Regulation” subscale (Hedge’s *g* = 0.58), reflecting that individuals with AN are more likely to notice and distract themselves from uncomfortable/painful body sensations, and are less likely to use self-regulation strategies. Participants with AN also reported lower scores on the “Body Listening” subscale compared to HC participants only, reflecting they are less likely to listen to their body signals/cues, (F(2,149) = 5.72; Hedge’s *g* = 0.92, CI [0.24–1.62]). Lastly, participants with AN reported lower scores on the “Trusting” subscale indicating difficulty experiencing their bodies as trustworthy (F(2,149) = 24.64; Hedge’s g > 0.80 compared to both ARFID (CI [− 1.91 to 0.41]) and HC participants (CI [− 1.26 to 2.6]).

### ARFID subgroup

In the entire ARFID sample (n = 30), no participants fell in the “Fear” subgroup exclusively. We used the NIAS and validated scoring guidelines and clinical cut-offs to assign group membership [31]. Our group membership was as follows: 12 participants in the “Sensory Sensitivity Eating” subgroup, 4 participants in the “Low Appetite” subgroup, 8 participants in the “Sensory Sensitivity Eating + Low Appetite” subgroup, 1 participant in the “Low Appetite + Fear of Aversive Consequences” subgroup, and 5 participants in all three subgroups. The Low Appetite subgroup had an interoceptive profile with the greatest accuracy (M = 0.83, SD = 0.14) and lowest awareness (r = 0.27, [−0.93 to 0.98]). The Sensory Sensitivity Eating subgroup appeared more consistent with the overall ARFID group, but this may also be because of the relatively larger sample (Accuracy M = 0.45 SD = 0.22; Awareness r = 0.55 [− 0.0 to 85]). Descriptive findings for all groups can be found in Fig. 1 and Table 3. For interoceptive sensibility, Body Trust was the highest in the Sensory Sensitivity and Low Appetite Groups, while Self-Regulation was lowest in the Low Appetite + Fear of Aversive Consequences Group (Fig. 2). Tests of significance or effect sizes were not run or generated due to small sample size and unequal distribution.

**Table 3:** Interoceptive awareness between ARFID subgroups

Subgroup	r	95% [CI]
Sensory sensitivity (n = 12)	0.55	− 0.04 to 0.85
Low appetite (n = 4)	0.27	− 0.93 to 0.98
Sensory sensitivity + low appetite (n = 8)	0.49	− 0.33 to 0.89
Low appetite + fear (n = 1)	–	–
Sensory sensitivity + low appetite + fear (n = 5)	0.26	− 0.81 to 0.93

Pearson’s *r* reflecting correlation between accuracy scores and confidence of accuracy guess

## Discussion

Overall, the results of this pilot study begin to shed light on interoceptive processes in adolescents with a restrictive eating disorder (AN and ARFID) and healthy comparisons. These data replicate existing adolescent norms using a multi-method battery of interoception and explore differences between two restrictive eating disorders for whom interoceptive functioning may relate to symptom development and expression [6]. In our sample, heartbeat guessing accuracy in healthy adolescents was found to be comparable to a sample of 80 adults completing the same task [27], consistent with prior research suggesting interoceptive accuracy may be established by adolescence [11]. Further, data show interoceptive awareness was slightly greater in a normative adult sample (*r* = 0.56) than in our adolescent sample (*r* = 0.35), indicating awareness of body signaling may continue to develop throughout adolescence. Lastly, for interoceptive sensibility in HC adolescents, our sample replicated the norms reported in the original validation study, highlighting that interoceptive sensibility continues to develop through adolescence [13].

With regards to interoceptive accuracy in clinical samples, it appeared that adolescents with ARFID *did* have poorer interoceptive accuracy (heartbeat detection) than HC participants, consistent with a priori hypotheses. However, ARFID participants’ confidence levels aligned with their heartbeat guesses, similar to HCs, suggesting that ARFID participants have intact metacognitive awareness of their interoceptive accuracy compared to AN participants, and similar to their healthy adolescent counterparts. These data may shed light on ARFID’s clinical picture: awareness of one’s reduced interoceptive accuracy may lead to increased caution around various aspects of eating, such as swallowing, eating quickly, or trying new foods.

Adolescents with AN had relatively higher interoceptive accuracy scores than those with ARFID (comparable to HC), which is partially consistent with our a priori hypotheses. However, their confidence did *not* map onto their accuracy, suggesting awareness of internal body sensations was relatively lower in AN participants relative to ARFID and HC groups. Prior research on interoceptive accuracy in AN has focused on adult samples, producing equivocal results [15, 44]. Consistent with our findings, one study of adults with AN reported no differences in heartbeat-tracking accuracy between AN participants and HCs, but found that participants with AN had lower interoceptive awareness [18].

Interoceptive sensibility, or self-reported interoception, on some subscales of the MAIA-Y varied between clinical cohorts and HC participants, aligned with hypotheses. Specifically, participants with ARFID reported difficulty not worrying about negative body sensations, using attention to body states to self-regulate distress, and trusting body signals. These findings are consistent with prior reports of ARFID participants viewing internal body sensations such as hunger or fullness as frightening, untrustworthy, or aversive [45] and identify a gap in this population’s ability to make sense of body cues and act adaptively. Individuals with AN reported a greater likelihood of ignoring uncomfortable body sensations and a lower likelihood of listening to or trusting their body cues. These findings may partially explain why interoceptive awareness was lower during the heartbeat guessing task: despite having relatively good accuracy, participants with AN have difficulty trusting and listening to body cues such as heartbeat—or more relevant to AN itself—gastric cues of hunger/satiety. However, individuals with AN may selectively attend to illness-specific cues, such as fullness and satiety, complicated by post-prandial discomfort in the context of slower digestion and gastric emptying [46].

Lastly, exploratory descriptive analyses within the ARFID group found relatively greater interoceptive accuracy in the Low Appetite subgroup than in other subgroups. The Low Appetite subgroup also had the lowest interoceptive awareness. This interoceptive profile looks similar to the AN group (e.g., greater accuracy and lower awareness). The exclusively Sensory Sensitivity subgroup had the lowest accuracy but the highest awareness, more characteristic of the ARFID subgroup as a whole. Regarding interoceptive sensibility, the Low Appetite and Sensory Sensitivity subgroups had greater body trust. Greater body trust may distinguish participants with Low Appetite ARFID participants from participants with AN, despite other domains of interoception looking similar. As prior research has shown, body trust reduces with age and negatively correlates with eating disorder symptoms. Thus, fostering body trust in adolescence may be a protective factor transdiagnostically [13, 19, 20]. Given the small sample size and unequal distributions, these interpretations should be read with caution.

This pilot study should be considered in light of several important limitations. First, due to this project's scope and limited funding, the first author completed all the assessments and was not masked to the diagnostic group. Future investigations benefit from masking assessors to reduce potential bias. Second, given ARFID’s heterogenous diagnostic categorization [3], interoception capabilities may vary by clinical presentation (fear, low appetite, and/or sensory sensitivity). We provide preliminary data probing ARFID subgroups; however, future research should consider larger samples to power investigations of interoception across different clinical presentations of ARFID. Next, as mentioned, the heartbeat tracking task can be influenced by factors such as BMI [47, 48] and sex assigned at birth [43], though both %EBW and sex were controlled for in current analyses. Future designs may be strengthened by matching participants for gender and sex. The heartbeat tracking task was used in this proof-of-concept study because it is a practical, low-cost, low-burden assessment procedure to interrogate interoception. Lastly, we recognize that the clinical sample sizes are small and preclude robust hypothesis testing. However, the results suggest that adolescents with restrictive eating disorders have differential interoceptive capabilities than healthy comparisons.

## Conclusions

The preliminary data reported here add to our understanding of interoception broadly and within restrictive eating disorders such as ARFID and AN. While this study is the first to our knowledge to explore interoceptive capabilities explicitly in youth with ARFID using a multi-method battery and exploring by subgroup, prior data suggest that these processes may be disrupted in this population. For example, painful and uncomfortable somatic sensations are common in children with ARFID, along with sensitivity to digestive cues [24, 49]. Future studies may consider using an interoceptive assessment that probes gastric cues, such as the water-loading task [50], for a more comprehensive and reliable interrogation of this population's interoceptive processes related to eating. The pilot data presented here are a first step in understanding how body sensations are understood and perceived in adolescent restrictive eating disorders compared to HCs.

## Data Availability

The datasets generated and analyzed during the current study are not publicly available due to privacy and ethical reasons but are available from the corresponding author on reasonable request.

## Abbreviations

ARFID: Avoidant/restrictive food intake disorder
AN: Anorexia nervosa
HC: Healthy controls
ED: Eating disorder
ES: Effect size
CI: Confidence interval
MAIA-Y: The multidimensional assessment of interoceptive awareness-2
